# Hepatitis B coinfection and awareness among patients with active tuberculosis in Georgia: a cross-sectional study

**DOI:** 10.1186/s12879-026-13816-3

**Published:** 2026-06-24

**Authors:** Giorgi Kanchelashvili, Jeffrey Collins, Zaza Avaliani, Maia Kipiani, Nestani Tukvadze, Mamuka Chincharauli, Lasha Gulbiani, Nino Bzishvili, George Kamkamidze, Maia Butsashvili

**Affiliations:** 1 Health Research Union, 8 Nutsubidze Street, Tbilisi, 0177 Tbilisi Georgia; 2 Emory University School of Medicine, Atlanta, GA USA; 3 European University, Tbilisi, Georgia; 4 National Center for Lung Health, Tbilisi, Georgia; 5 The University of Georgia, Tbilisi, Georgia; 6 Swiss Tropical and Public Health Institute, Allschwil, Switzerland; 7 Clinic NeoLab, Tbilisi, Georgia

**Keywords:** Tuberculosis, Hepatitis B virus, Coinfection, Vaccination, Georgia

## Abstract

**Background:**

Patients with TB disease in Georgia are not routinely tested for HBV and the prevalence of chronic HBV among TB patients is unknown. We aimed to assess the prevalence of HBV coinfection and immunity among persons with TB in Georgia and evaluate their HBV-related knowledge and attitudes toward vaccination.

**Methods:**

We conducted a cross-sectional study among persons with active TB disease at the National Center for Tuberculosis and Lung Diseases in Georgia from September 20, 2024 to February 20, 2025. Participants underwent serologic testing (HBsAg, anti-HBs, anti-HBc) and completed a questionnaire on HBV knowledge, vaccination history, and willingness to vaccinate.

**Results:**

We enrolled 110 participants (median age = 44.0, IQR:35.0-56.3), of which 63.6% were male. Four participants (3.6%) were HBsAg positive. HBV test results showed 14 (12.7%) had past HBV infection with anti-HBc+/anti-HBs+, and 7 (6.4%) had isolated anti-HBc positivity (anti-HBc+/anti-HBs-). Another seven (6.4%) had vaccine-induced immunity (anti-HBc-/anti-HBs+), and 82 (74.5%) were susceptible to HBV infection. While 74.5% reported having heard of HBV, only 50.0% correctly identified transmission routes. Only 16 patients (14.5%) were willing to receive vaccination. Among those unwilling, the main barriers were concerns about vaccine safety (34.9%), effectiveness (23.3%), and perceived infection risk (20.9%). Participants aged ≤ 40 years were significantly more likely to be aware of HBV compared to those over 40 (OR=3.39, 95% CI: 1.25–9.20).

**Conclusion:**

TB-HBV coinfection observed in this study suggests that routine HBV screening among TB patients in Georgia may be beneficial. Low vaccination coverage and limited knowledge highlight the importance of integrating HBV education and vaccination into TB programs to improve outcomes.

**Supplementary Information:**

The online version contains supplementary material available at https://doi.org/10.1186/s12879-026-13816-3.

## Introduction

Tuberculosis (TB) and hepatitis B virus (HBV) are two major infectious diseases that continue to pose substantial challenges to global public health. According to the World Health Organization (WHO), TB ranks as the leading cause of death from infectious disease globally, with an estimated 10.8 million new cases in 2023 [1]. Despite substantial progress in TB control over the past decade, TB remains a major public health issue in the country of Georgia. The National TB Program has achieved notable success in improving diagnostic and treatment services, with an overall decline in TB incidence as estimated by WHO over the past 15 years [2, 3]. In 2023, WHO estimated that Georgia had a TB incidence of 55 (44–69) cases per 100,000 population, corresponding to 2,100 incident cases [4]. Since 2016, Georgia has no longer been classified by WHO as a high-burden country for multidrug-resistant tuberculosis, largely due to improvements in treatment approaches, including the introduction of newer medications [3].

In 2022, 254 million people globally were living with chronic HBV infection, and approximately 1.1 million deaths occurred due to complications such as cirrhosis and hepatocellular carcinoma [5]. Data from the 2021 national serosurvey in Georgia indicate an anti-HBc prevalence of 21.7% and an HBsAg prevalence of 2.7%, which was largely unchanged from prior surveys [6]. Vaccination remains one of the most effective measures for HBV prevention [7]. However, while childhood vaccination coverage in Georgia has exceeded 90% since 2010, vaccine coverage remains poor in older cohorts with important comorbidities. Only 1.1% of patients with chronic HCV reported ever having been vaccinated against HBV, and coverage remains low among people who inject drugs (PWID). Public surveys conducted over the last decade have shown most people in Georgia (> 63%) are unaware of HBV, and even among those who are aware, many do not know that treatment options exist for chronic infection [6]. Thus, HBV continues to pose a considerable health burden in Georgia.

Although TB and HBV differ in their transmission routes and clinical characteristics, they often affect the same populations, creating important clinical implications in cases of coinfection, such as a higher risk of drug-induced liver injury from TB treatment and the need for routine HBV screening and close liver function monitoring in TB patients [8, 9]. In the United States, latent tuberculosis infection is more than twice as common in patients with chronic HBV infection, suggesting that these patients are at higher risk of TB infection than the general population [9]. Furthermore, HBV and TB co-infection appears to be associated with an increased risk of drug-induced liver injury (DILI) during TB therapy, underscoring the urgent need to diagnose and treat those with chronic HBV who are treated for TB. HBV-TB coinfection has also been associated with poorer treatment outcomes, particularly due to an increased risk of drug-induced liver injury during anti-tuberculosis therapy, which may lead to treatment interruptions and complications [10].

In Georgia, Tuberculosis services are delivered through a centralized national program, with specialized institutions such as the National Center for Tuberculosis and Lung Diseases (NCTLD; now known as the National Center for Lung Health (NCLH)) playing a key role in diagnosis, treatment, and follow-up of patients. A substantial proportion of patients receive care in outpatient settings, particularly during the continuation phase of TB treatment, making ambulatory clinics an important point of contact for integrated screening and preventive interventions [11, 12].

Little is known about the prevalence of chronic HBV infection among people who develop TB disease either globally or in Georgia. We conducted this study to determine the prevalence of HBV coinfection and serologic evidence of HBV immunity (from past infection or vaccination) among patients with active TB in Georgia, and to assess their HBV-related knowledge, vaccination history, and willingness to receive HBV vaccination.

## Methods

### Study design, setting and eligibility criteria

We conducted a cross-sectional study among adults with any form of active TB disease receiving treatment at the NCTLD in Tbilisi, Georgia. Participants were consecutively enrolled from the outpatient department during the study period, from September 20, 2024, to February 20, 2025. Eligible participants were adults (≥ 18 years) receiving treatment for any form of active TB (pulmonary or extrapulmonary; bacteriologically confirmed or clinically diagnosed), and able and willing to provide informed consent and complete the questionnaire in the local language (Georgian). All forms of extrapulmonary TB were included, and cases were not further classified by anatomical site. We excluded individuals < 18 years and individuals unable to provide informed consent (e.g., due to severe illness or cognitive impairment). No additional exclusion criteria were applied, and the only individuals not included in the study were those who declined participation.

### Sample size and data collection

In 2023, a total of 295 patients with any form of active TB received outpatient care at NCTLD, while in 2024 this number was 269. We did not perform a formal sample size calculation. Based on the annual outpatient TB caseload at NCTLD and given the available resources and one-year project timeline with a planned five-month prospective enrolment period, we aimed to consecutively enroll all eligible TB patients presenting during the study period.

Face-to-face interviews in Georgian were conducted by treating physicians using a structured, interviewer-administered questionnaire consisting of 21 closed-ended questions on socio-demographic data, HBV vaccination history, willingness to receive vaccination, and knowledge about HBV symptoms, transmission routes, treatment, and vaccination. The questionnaire was developed based on a previously used instrument from a knowledge, attitudes, and practices (KAP) study on hepatitis B conducted in Georgia in 2023 (Incentivized Peer Interventions to Support Hepatitis B Vaccination among People Who Inject Drugs in Georgia). The questionnaire was adapted and modified by the research team to fit the objectives of the current study. An English-language version of the questionnaire has been provided as supplementary material (Additional file 1).

### Laboratory methods

A 5 mL venous blood sample was collected from each participant by a trained nurse for laboratory testing of hepatitis B surface antigen (HBsAg), hepatitis B surface antibody (anti-HBs), and hepatitis B core antibody (anti-HBc). Blood samples were stored at NCTLD under refrigeration according to standard guidelines and transported every two days to the Clinic NeoLab (Tbilisi, Georgia) for testing. At Clinic NeoLab, serologic testing for HbsAg, anti-HBs, and anti-HBc was performed using chemiluminescent immunoassays (CMIA) on the ARCHITECT system (Abbott Laboratories, USA), following the manufacturers’ instructions and routine internal quality control procedures. Laboratory procedures adhered to standard operational protocols for sample handling and transport. Participants with positive HBsAg were referred to hepatitis services for evaluation and treatment in accordance with national practice.

### Ethical considerations

All study data were pseudonymized before analysis, and no directly identifying personal information was retained in the study dataset. The study was approved by the Institutional Review Boards of the Health Research Union (IRB00009520; IORG0005619), the National Center for Tuberculosis and Lung Diseases (IORG0009467; FWA00020831), and Emory University (IRB ID: STUDY00007701). The research was conducted in compliance with the Georgian legislation, U.S. Department of Health and Human Services regulations (45 CFR 46), and relevant international guidelines for biomedical research. The study was conducted in accordance with the Declaration of Helsinki and its later amendments. All participants provided written informed consent in the Georgian language before any study procedures were initiated.

### Statistical analysis

Statistical analyses were performed using SPSS v.26.0 (Chicago, IL). Descriptive statistics summarized participant characteristics, HBV serological results, vaccination status, and HBV-related knowledge and attitudes. Associations between categorical variables were examined using the Chi-square test. Odds ratios (ORs) with 95% confidence intervals (CIs) were calculated for selected associations. A p value < 0.05 was considered statistically significant. Multivariable analysis was not performed because of the small sample size and limited number of outcome events.

## Results

We invited 124 eligible patients to participate in the study, of whom 110 (88.7%) agreed and were enrolled. No demographic or clinical data were collected from individuals who declined participation. A total of 110 participants were enrolled in the study, of which 63.6% (*n* = 70) were male. The median age was 44.0 years (IQR: 35.0-56.3). The majority of participants (60.0%, *n* = 66) were older than 40 years. Most participants resided in urban areas (90.0%, *n* = 99). Half of the participants (50.9%, *n* = 56) had secondary education, 42.7% (*n* = 47) vocational education, and 6.4% (*n* = 7) higher education. The majority (93.6%, *n* = 103) were of Georgian ethnicity. Employment status varied, with 44.5% (*n* = 49) employed, 39.1% (*n* = 43) temporarily unemployed, 10.0% (*n* = 11) pensioners, 4.5% (*n* = 5) housewives, and 1.8% (*n* = 2) students.

Most participants had newly diagnosed drug-susceptible TB disease, and pulmonary TB was the most common presentation. Four participants were HBsAg-positive, and none were previously aware of their infection. HBV test results showed that anti-HBc positivity was detected in 19.1%. Among them, 14 patients had past HBV infection with anti-HBc+/anti-HBs+, and 7 participants had isolated anti-HBc positivity (anti-HBc+/anti-HBs-). Another seven participants had vaccine-induced immunity (anti-HBc-/anti-HBs+), while 82 were susceptible to HBV infection (Table 1).

**Table 1:** TB disease characteristics and HBV serologic results (Total = 110)

Characteristics	*N*	%
TB case classification by treatment history		
New	98	89.1
Previously treated	12	10.9
TB case classification by drug-resistance status		
Drug-susceptible TB	95	86.4
Any drug-resistant TB	15	13.6
Site of TB disease		
Pulmonary	91	82.8
Extrapulmonary	16	14.5
Both	3	2.7
HbsAg results		
Positive	4	3.6
Negative	106	96.4
Anti-HBs results		
Positive (Anti-HBc positive)	14	12.7
Positive (Anti-HBc negative)	7	6.4
Negative	89	80.9
Anti-HBc results		
Positive	21	19.1
Negative	89	80.9

Abbreviations: TB = tuberculosis; HBV = hepatitis B virus; HBsAg = hepatitis B surface antigen; anti-HBs = hepatitis B surface antibody; anti-HBc = hepatitis B core antibody

Participants were asked whether they had ever heard of hepatitis B, and 73.6% (*n* = 81) responded that they had. Only 50.0% (*n* = 55) of all participants knew at least two transmission routes of hepatitis B. The most frequently recognized modes of transmission included blood transfusion (50.9%, *n* = 56), used needle/syringe (34.5%, *n* = 38), and receiving medical/dental services (32.7%, *n* = 36). Less commonly reported routes were unprotected sex (17.3%, *n* = 19), sharing personal hygiene products (4.5%, *n* = 5), and from mother to child during childbirth (5.5%, *n* = 6). Notably, few participants incorrectly identified food and water (5.5%, *n* = 6) and airborne transmission (2.7%, *n* = 3) as possible routes. Overall, 41.8% (*n* = 46) did not know any transmission route.

Only 12.7% of participants (*n* = 14) reported that they had been tested for HBV. Among them, 78.6% (*n* = 11/14) reported a negative result, 7.1% (*n* = 1/14) - active infection, and 14.3% (*n* = 2/14) - a previous infection. Only 16.4% (*n* = 18) believed there is an effective treatment for HBV.

Knowledge about the existence of a vaccine and actual vaccination coverage were very low, while willingness to be vaccinated was limited. The majority reported insufficient information about HBV, though most expressed interest in receiving more, with the internet and health workers being the preferred sources (Table 2).

**Table 2:** HBV vaccination status, willingness to receive HBV vaccination, knowledge, and information sources among TB patients in Georgia

Characteristics	*N*	%
Is there a hepatitis B vaccine? (*N* = 110)		
Yes	10	9.1
No	1	0.9
I Don’t know	99	90.0
Have you been vaccinated against hepatitis B? (*N* = 110)		
Yes	2	1.8
No	104	94.5
I Don’t remember	4	3.6
If no or don’t know, would you take the hepatitis B vaccine if offered? (*N* = 108)		
Yes	16	14.8
No	41	38.0
I Don’t know	45	41.7
Refused to answer	6	5.6
If no, what is the main reason? (*N* = 41)		
I do not believe in the effectiveness of the vaccine	9	22.0
The vaccine may cause other complications	15	36.5
You may be infected with the hepatitis B virus from the vaccine	9	22.0
I don’t know	3	7.3
Refusal to answer	5	12.2
Do you think you have enough information about hepatitis B?		
Yes	6	5.5
No	87	79.1
I don’t know/I’m not sure	17	15.5
Would you like to receive more information about hepatitis B? (*N* = 110)		
Yes	97	88.2
No	13	11.8
Which of the following would be the best source of information for you about hepatitis B? (*N* = 110)		
Television	30	27.3
Radio	4	3.6
Magazines and newspapers	12	10.9
Internet	82	74.5
Special medical literature	21	19.1
Health worker	54	49.1

Note: Denominators vary across questions due to conditional responses

Only 2 participants reported having been vaccinated against HBV, and serological testing confirmed that only 1 of them was anti-HBs positive. In contrast, 7 participants had serological evidence of vaccine-induced immunity, indicating a discrepancy between self-reported and serologically confirmed vaccination status.

### Bivariate association between HBV knowledge and demographic variables

Bivariate analysis showed that, compared with respondents who had complete secondary education, those with vocational/higher education were more often ≤ 40 years old, more frequently reported having heard of hepatitis B, more often perceived HBV as a serious health problem for Georgia, more commonly believed HBV to be contagious and more frequently identified at least two correct transmission routes (Table 3).

**Table 3:** Bivariate associations between education level and HBV-related knowledge and attitudes

Characteristics	Education		OR (95% CI)	*p* value
	Complete secondary*	Vocational/ higher education		
Age groups				
≤ 40 years	17 (38.6%)	27 (61.4%)	2.29 (1.05–5.01)	0.036
> 40 years	39 (59.1%)	27 (40.9%)	1	
Have you ever heard of a disease called hepatitis B?				
Yes	36 (64.3%)	45 (83.3%)	2.78 (1.13–6.84)	0.023
In your opinion, how serious is hepatitis B for Georgia?				
A serious problem	15 (26.8%)	36 (66.7%)	5.47 (2.41–12.39)	< 0.001
Do you think hepatitis B is a contagious disease?				
Yes	22 (39.3%)	35 (64.8%)	2.85 (1.31–6.18)	0.007
Knew at least two correct transmission routes of hepatitis B*				
Yes	21 (37.5%)	34 (63.0%)	2.33 (1.03–5.29)	0.008

Abbreviations: OR= odds ratio; CI= confidence interval; HBV = hepatitis B virus

For all characteristics except age group, the reference category was complete secondary education

* This variable was created from the multiple-choice question about transmission routs. Participants who correctly identified two or more hepatitis B transmission routes were coded as “Yes”

Note: Results are based on bivariate analysis. ORs are unadjusted

Patients aged > 40 years (25.8%) were significantly more likely to have anti-HBc positive result compared to those aged ≤ 40 years (9.1%) (OR = 3.47; 95% CI: 1.08–11.14). Participants aged ≤ 40 years were significantly more likely to be knowledgeable about HBV compared to those over 40 (OR = 3.39, 95% CI: 1.25–9.20).

## Discussion

In this study, all identified HBsAg positive cases represented previously unrecognized infection. Protection against hepatitis B was limited, with low reported vaccination. These findings highlight the importance of systematic HBV case finding in TB settings and support routine HBV screening with vaccination and linkage to care within TB services.

The prevalence of HBsAg positivity in our study was higher than the 2.7% observed in the 2021 national survey of the adult Georgian population [6]. However, this estimate is based on a relatively small sample size (*n* = 110) and should be interpreted with caution. In addition, differences in demographic characteristics, particularly age distribution, as well as the clinical nature of the study population, may partly explain the observed difference. Notably, those patients were unaware of their hepatitis B infection, underscoring missed opportunities for case-finding in TB settings. These findings suggest that routine HBV screening among TB patients may be considered to better understand the burden of coinfection and to facilitate timely linkage to care for those with chronic infection. WHO recommends that in settings with HBsAg prevalence above 2%, all adults be offered HBsAg testing with linkage to prevention, care, and treatment; this supports implementing routine testing within TB services [5]. Patients with TB may face additional challenges in recognizing and addressing HBV risk due to competing health priorities, stigma, and limited provider-patient communication about HBV.

Our study identified substantial gaps in hepatitis B awareness and vaccination among patients with active tuberculosis in Georgia. Most participants had little or no knowledge of the disease, its modes of transmission, or the existence of a vaccine. Even more concerning was the lack of willingness to be vaccinated. An important finding of our study is the discrepancy between self-reported and serologically confirmed vaccination. Although 7 participants had serological evidence of vaccination, only 1 of them correctly reported being vaccinated. This mismatch highlights a considerable lack of knowledge and awareness regarding HBV vaccination status among TB patients. This discrepancy may also be explained by failure to develop a protective immune response or receipt of an incomplete vaccination series. The fact that some participants who were vaccinated did not recall their status suggests that HBV vaccination is not perceived as important, further underlining the low level of awareness and engagement with HBV prevention in this population. This knowledge gap represents a significant public health concern because individuals with active TB are at elevated risk for HBV coinfection, and prevention through vaccination is both available and effective [13, 14].

The results of our bivariate analysis highlight the central role of education. Participants with vocational or higher education were more likely to have heard of HBV, to perceive it as a serious health problem for Georgia, to believe it is contagious, and to correctly identify at least two routes of transmission. Our data also showed that younger participants were more likely to have higher educational attainment, which may partly explain why they demonstrated better knowledge and awareness of HBV compared with older participants. Despite the low level of HBV knowledge observed in our sample, a study conducted among patients with active TB in Myanmar reported higher levels of awareness, with 44.5% of participants demonstrating knowledge about hepatitis B and 87% recognizing that HBV is a contagious disease [15]. This difference may reflect variations in local health system organization, patient education practices, and integration of HBV-related information into TB care services. In contrast, other studies conducted among high-risk populations, including people who inject drugs and healthcare workers, have also reported low levels of HBV knowledge and limited awareness of transmission routes and prevention strategies [16–18]. These findings suggest that HBV awareness varies considerably across populations and settings, likely influenced by differences in public health programming, access to information, and targeted education efforts. In our study, the low level of knowledge observed among TB patients highlights the need for improved patient education, particularly within TB care settings, where opportunities for integrated screening and prevention may exist.

This study has several limitations that should be considered when interpreting the findings. First, it was conducted at a single specialized referral center (NCTLD) and included patients receiving care in an ambulatory setting. Therefore, the findings represent a facility-based sample and may not be generalizable to all patients with TB in Georgia. Second, the sample size was relatively small and determined by the study budget, timeline, and the use of consecutive sampling, which may limit the precision of the estimates and the ability to detect smaller associations. In addition, analyses were limited to bivariate comparisons, and multivariable modeling was not performed because of the small sample size and limited number of outcome events. Third, the cross-sectional design precludes drawing causal inferences between variables, and the observed relationships should be interpreted as associations only. Finally, HBV serological status was assessed at a single time point, which limits the ability to distinguish between acute, chronic, or resolved infections.

One of the strengths of this study is that, to our knowledge, it is the first to assess HBV prevalence, immunity, and knowledge among patients with active TB in Georgia. The results provide insight into the overlap of two important infectious diseases within a high-risk group. These findings may help guide national HBV elimination efforts and TB control activities by supporting more coordinated approaches to prevention, screening, and treatment.

## Recommendations and conclusion

These findings have several important implications for public health in Georgia. First, TB services might consider integrating educational interventions into TB care to address knowledge gaps about HBV, with tailored messages for patients of different educational backgrounds. Second, HBV testing may be offered to all TB patients, and those testing negative for antibodies might be provided on-site vaccination during TB treatment. However, it should be noted that vaccine immunogenicity may be reduced during active TB disease, and TB patients represent a relatively small proportion of the overall HBV burden. Therefore, this approach should be considered as a targeted, programmatic intervention rather than a primary population-level strategy. Given that HBV vaccination is already free in Georgia [19], integrating this service into TB clinics may be feasible and could improve uptake. Third, patients with active TB might be considered for routine HBsAg testing as part of their initial clinical evaluation. Early identification of HBV infection would enable timely linkage to appropriate care. Patients testing positive could be referred directly to the free state HBV treatment program, available since 2024 [19]. This integrated approach may help prevent HBV transmission and potentially lower the risk of severe liver disease and drug-induced liver injury during TB therapy [13].

In conclusion, our findings suggest that TB-HBV coinfection may represent an important public health concern in Georgia. The higher HBV prevalence observed among TB patients in this study, combined with low awareness and vaccination coverage, highlights potential gaps in prevention and care. Integrating HBV education, testing, and on-site vaccination into TB care could reduce the risk of coinfection, prevent further HBV transmission, and improve long-term health outcomes.

## Supplementary Information

Below is the link to the electronic supplementary material.

Supplementary Material 1

## Data Availability

The datasets used and/or analysed during the current study are available from the corresponding author on reasonable request.
