# Supplementary material for: Hepatitis B coinfection and awareness among patients with active tuberculosis in Georgia: a cross-sectional study
Source: BMC Infect Dis. 2026 Jun 24;26:1625. doi: 10.1186/s12879-026-13816-3 (PMC13560259; doi:10.1186/s12879-026-13816-3)
Supplement: Supplementary file 1 [file 12879_2026_13816_MOESM1_ESM.pdf]

Epidemiology of TB-HBV coinfection and evaluation of willingness of HBV vaccination among patients with active TB in Georgia

Questionnaire

Completion date:

Participant ID:

Interviewer:

TB treatment start date:

Classification of a person with tuberculosis according to their history of previous anti-TB treatment:

1. New 2. Previously treated 3. Unknown

Classification of a person with tuberculosis according to susceptibility or resistance to anti-TB medications:

1.Drug-susceptible TB (DS-TB) 2. Drug-resistant TB (DR-TB)

Localization of the TB disease process:

1. Pulmonary 2. Extrapulmonary 3. Both: pulmonary and extrapulmonary

E-mail for results: ____

N	Question	Answer
1	Age	___________
2	Sex	1.Male 2. Female
3	Address	Region ------------------------<br>City/Town --------------------------
4	Type of residence	1. Urban<br>2. Rural
5	Marital status	1.Single<br>2.Married<br>3.Divorced<br>4.Widow
6	Level of education	1.Incomplete secondary<br>2. Complete secondary<br>3.Vocational<br>4.Incomplete higher education<br>5.Complete higher education<br>99. Did not answer
7	Nationality	1. Georgian<br>2. Other______________

		99. Did not answer
8	Employment status	1. Never worked<br>2. Temporarily unemployed<br>3. Employed<br>4. Student/school pupil<br>5. Pensioner<br>6. Housewife<br>7. Other________________________
9	Have you ever heard of a disease called hepatitis B?	1. Yes<br>2. No *(skip to question ##)*
10	In your opinion, how serious is hepatitis B for Georgia? **(One answer only)**	1. A serious problem<br>2. Not so serious problem<br>3. No problem at all<br>88. I don't know<br>99. Did not answer
11	Do you think hepatitis B is a contagious disease?	1. Yes<br>2. No *(skip to question ##)*<br>3. Don't know *(skip to question ##)*
12	In your opinion, how is hepatitis B transmitted from person to person? **(Several answers are possible)**	1. Through food and water<br>2. By blood transfusion<br>3. By air<br>4. Unprotected sex<br>5. By shaking the hand of a sick person<br>6. By receiving medical/dental services<br>7. Using a used needle/syringe<br>8. From mother to child during pregnancy<br>9. From mother to child during childbirth<br>10. By sharing personal hygiene products<br>11. Other (specify)____________________________________<br>88. I don't know<br>99. Did not answer
13	Have you ever been tested for hepatitis B?	1. Yes<br>2. No *(skip to question ##)*<br>88. I don't remember *(skip to question ##)*<br>99. Refusal to answer *(skip to question ##)*
14	If yes, what was the result?	1. Negative<br>2. Positive, active infection<br>3. Positive, previously transmitted form of infection<br>88. I don't remember<br>99. Refusal to answer
15	Do you think there is an effective treatment for hepatitis B?	1. Yes<br>2. No<br>88. I don't know<br>99. Refusal to answer
16	Is there a hepatitis B vaccine?	1. Yes<br>2. No

		88. I don't know<br>99. Refusal to answer
17	Have you been vaccinated against hepatitis B?	1. Yes *(skip to question ##)*<br>2. No<br>88. I don't know<br>99. Refusal to answer *(skip to question ##)*
18	If no or don't know, would you take the hepatitis B vaccine if offered?	1. Yes *(skip to question ##)*<br>2. No<br>88. I don't know *(skip to question ##)*<br>99. Refusal to answer *(skip to question ##)*
19	If no, what is the main reason?	1. I do not believe in the effectiveness of the vaccine<br>2. it`s expensive<br>3. I don't have enough time<br>4. The vaccine may cause other complications<br>5. You may be infected with the hepatitis B virus from the vaccine<br>88. I don't know<br>99. Refusal to answer
20	Do you think you have enough information about hepatitis B?	1. Yes<br>2. No<br>88. I don't know/I'm not sure<br>99. Did not answer
21	Would you like to receive more information about hepatitis B?	1. Yes<br>2. No<br>88. I don't know/I'm not sure<br>99. Did not answer
22	Which of the following would be the best source of information for you about hepatitis B? **(Several answers are possible)**	1. Television<br>2. Radio<br>3. Magazines and newspapers<br>4. Internet<br>5. Friends/relatives<br>6. Special medical literature<br>7. Health worker<br>8. Other (specify) ____________________<br>88. I don't know<br>99. Did not answer
